# Obstetric Cholestasis among Pregnant Women Visiting a Tertiary Care Centre

**DOI:** 10.31729/jnma.8283

**Published:** 2023-10-31

**Authors:** Indra Yadav, Siddhartha Kumar Yadav, Tarun Pradhan, Anika Yadav, Sabita Jyoti, Rozy Yadav

**Affiliations:** 1 Department of Obstetrics and Gynaecology, Birat Medical College and Teaching Hospital, Biratnagar, Morang, Nepal; 2 Department of Paediatrics, ChitWan Medical College, Bharatpur, Chitwan, Nepal; 3 Department of Community Medicine, Nepalgunj Medical College Teaching Hospital, Nepalgunj, Banke, Nepal; 4 Department of Obstetrics and Gynaecology, Karnali Province Hospital, Birendranagar, Sublet, Nepal

**Keywords:** *Nepal*, *obstetric cholestasis*, *pregnancy*

## Abstract

**Introduction:**

Obstetric cholestasis is one of the most frequent hepatic disorders specific to pregnancy. It occurs commonly during the latter half of pregnancy. The data regarding this issue is rare in our settings. The aim of this study was to find out the prevalence of obstetric cholestasis among pregnant women visiting a tertiary care centre.

**Methods:**

A descriptive cross-sectional study was conducted among pregnant women, who attended a tertiary care centre from 24 July 2022 to 24 January 2023. Ethical approval was taken from the Institutional Review Committee of the same institute. Convenience sampling method was used. The point estimate was calculated at a 99% Confidence Interval.

**Results:**

The prevalence of obstetric cholestasis was 80 (1.38%) (1-1.80, 99% Confidence Interval). A total of 40 (50%) were in the age group 21-30 years, with a mean age of 28.06±6.39 years. A total of 48 (60%) were multigravida and 73 (91.30%) had singleton pregnancies. Pruritus of the whole body was complained of by 56 (70%) patients. Previous history of obstetric cholestasis was present in 21 (26.30%).

**Conclusions:**

The prevalence of obstetric cholestasis among pregnant women is higher than other studies done in similar settings.

## INTRODUCTION

Obstetric cholestasis (OC) is one of the most frequent hepatic disorders specific to pregnancy. It occurs commonly during the latter half of pregnancy. OC is also known as intrahepatic cholestasis of pregnancy (ICP) and is a reversible and reoccurring type of hormonally influenced cholestasis.^1^ Incidence varies from 0.1 to 15.6% depending on geography and ethnicity. The incidence of ICP among Indian women reported is around 1% and in Eastern Nepal 1.15%.^2^ The prevalence of overall ICP is around 0.1-2%, whereas in the Asian Indian population, it is around 1.2-1.5%.^3-5^

OC is a stressful condition for mothers and is associated with significant maternal morbidities.^6,7^ There are limited studies regarding this issue. Data regarding this issue may aid in the prevention of further complications regarding the disease.

The aim of this study was to find out the prevalence of obstetric cholestasis among pregnant women visiting a tertiary care centre.

## METHODS

This descriptive cross-sectional study was conducted among pregnant women presenting to the Birat Medical College and Teaching Hospital (BMCTH), Biratnagar, Morang, Nepal from 24 July 2022 to 24 January 2023. Ethical approval was obtained from the Institutional Review Committee (Reference number: IRC-PA-213/2078-79) of the same institute. Pregnant women visiting to the BMCTH during the study period were included. Those women who are positive for hepatitis, present history of gallbladder disease, hypertension complicating pregnancy, autoimmune disease like primary biliary cirrhosis and also those with a history of any dermatological problem were excluded. Written informed consent was taken from participants. The convenience sampling method was used. The sample size was calculated using the following formula:


$$\begin{array}{ll} \text{n} & ={\text{Z}}^{2}\times \frac{\text{p}\times \text{q}}{{\text{e}}^{\text{2}}}\\ & =2.57^{2}\times \frac{0.025\times 0.975}{0.01^{2}}\\ & =1610 \end{array}$$

Where,

n = minimum required sample size
Z = 2.57 at 99% Confidence Interval (CI)
p = prevalence taken as 2.5% from the previous study^8^
q = 1-p
e = margin of error, 1%

The calculated sample size was 1610. By tripling the sample size, the calculated sample size was 4,830. However, 5,800 pregnant women were evaluated. Among them, 80 were excluded as per exclusion criteria. So, a total of 5,780 women were included in the study. Face-to-face interviews were conducted, and different parameters of the questionnaire were asked including aggravating and relieving factors. They were followed up till 15-30 minutes after delivery and their maternal and fetal consequence were recorded. At the time of delivery, the weeks of gestation, the onset of labour and the mode of delivery were noted and the intrapartum complications were observed. The maternal and fetal issues were observed and recorded.

The liver function test (LFT) was repeated every 2-4 weeks intervals as required and on the basis of the severity of the case. Hemoglobin, fasting lipid profile and coagulation profile were also done. In the present study, serum levels of aminotransferase, bilirubin, etc. that are more than the upper limit of pregnancy-specific reference ranges are considered positive for OC. However, measurement of serum bile acid could not be done. All patients included in the study were given ursodeoxycholic acid (UDCA) 150-900 mg/day in divided doses for the rest of the antenatal period.^9^

The data collected were entered in Microsoft Excel 2010 and analyzed using IBM SPSS Statistics version 22.0. The point estimate was calculated at a 99% CI.

## RESULTS

Among 5720 pregnant women, the prevalence of OC was 80 (1.38%) (1-1.80, 99% CI). The mean age was 28.06±6.39 years with a median age of 27 years. The mean gestational period was 31.38±4.56 weeks and the median was 32 weeks. A total of 66 (82.50%) of them were born at term and 13 (16.25%) were born at preterm, however, only 1 (1.25%) baby was born postdated (Table 1).

**Table 1:** Demographic distribution of pregnant women with OC (n= 80).

Parameters	n (%)
**Age (years)**	
≤20	7 (6.90)
21-30	40 (50)
31-40	32 (40)
>40	1 (1.25)
**Body mass index (kg/m^2^)**	
Underweight (<18.50)	1 (1.25)
Normal (18.50-24.99)	64 (80)
Overweight (25-29.99)	15 (18.75)
**Gravida**	
Primigravida	28 (35)
Multigravida	48 (60)
Grand multipara	4 (5)
**Period of gestation (weeks)**	
14-26	8 (10)
27-40	72 (90)
**Type of pregnancy**	
Singleton pregnancies	73 (91.25)
Multifetal pregnancies	7 (8.75)
**Timing of delivery**	
Preterm	13 (16.25)
Term	66 (82.50)
Post-dated	1 (1.25)

The most frequent chief complaints were pruritus of the whole body in 56 (70%) followed by pruritus of palm and sole which worsened at night in 24 (30%). A previous history of similar complaints was seen in 21 (26.25%) patients. However, 6 (7.50%) women had a family history of OC (Table 2).

**Table 2:** LFT parameters of pregnant women with OC (n= 80).

Parameters	n (%)
Alanine aminotransferase (IU/L)	67 (83.75)
Aspartate aminotransferase (IU/L)	67 (83.75)
Alkaline phosphatase (IU/L)	49 (61.25)
Total bilirubin (mg/dl)	28 (35)

**Figure 1:**
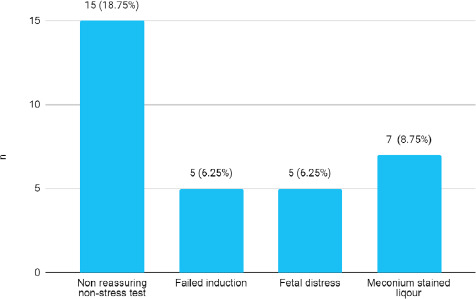
Indication of emergency LSCS (n= 80)

A total of 45 (56.25%) patients were given UDCA tablet 150 mg twice a day followed by 26 (32.50%) who were given UDCA 300 mg twice a day while 9 (11.25%) with UDCA 300 mg thrice a day. The majority of women 68 (85%) had lower section cesarean section (LSCS), 32 (40%) had emergency LSCS and 36 (45%) had elective LSCS. However, in this study 12 (15%) of women had vaginal delivery which included vaccum delivery 2 (2.50%), spontaneous vaginal delivery 5 (6.25%) and and out of 27 induced cases only 5 (6.25%) patients had vaginal delivery. Indications of emergency LSCS were non-reassuring non-stress test 15 (18.75%) and failed induction 5 (6.25%) (Figure 1).

**Figure 2:**
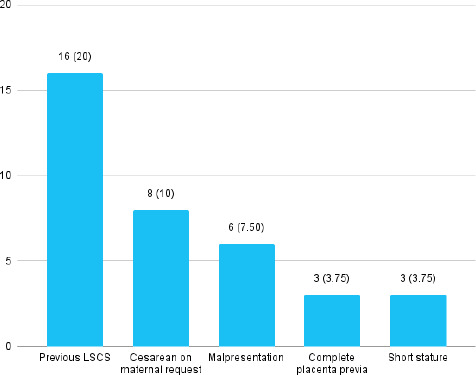
Indication of elective LSCS (n= 80).

The indication for elective LSCS was previous LSCS in 16 (20%) and maternal request in 8 (10%) (Figure 2).

The complications encountered following LSCS and vaginal delivery were postpartum haemorrhage (PPH) seen in 13 (16.25%), coagulation disorder in 7 (8.75%), and surgical site infection (SSI) in 1 (1.25%).

Birth weight of the majority 57 (71.25%) of newborns was within the normal range (2500-3500 gm) followed by 22 (27.50%) of low birth weight (1000-2500 gm) and 1 (1.25%) with very low birth weight (<1000 gm). Similarly, more than half 42 (52.50%) had scored 6 at 1 minute and 58 (72.50%) had scored 8 at 5 minutes respectively. The APGAR scores at 1 minute were a maximum score of 8, a minimum of 6 with a mean score of 6.58±0.45, a median of 6 and at 5 minutes APGAR scores were a maximum score of 9 and a minimum of 6 with a mean score of 7.79±0.65, a median 8 (Table 3).

**Table 3:** APGAR score of newborns of women with OC (n= 80).

APGAR score at 1 min	n (%)
6	42 (52.50)
7	30 (37.50)
8	8 (10)
**APGAR score at 5 min**	
6	5 (6.25)
7	12 (15)
8	58 (72.50)
9	5 (6.25)

The complications encountered by neonates were NICU admission 37 (46.25%), transient tachypnea of the newborn (TTN) 12 (15%), meconium aspiration syndrome 8 (10%), meconium stained liquor 4 (5%) and neonatal jaundice 2 (1.50%).

## DISCUSSION

In obstetrics practice, OC is relatively common, with no exact know factor but thought to be multifactorial with few of recognized factors are genetic configuration, environmental changes, ethnicity also the geographical areas.^9^ In this study, the prevalence of obstetric cholestatic among pregnant women was found to be 1.38% that is lesser than the similar studies done by other authors (2.5%),^8^ (3.3%)^9^ respectively. However, higher than the study in eastern part of the Nepal (1.15%), the difference in prevalence could be because of the variance in sample size as well as settings.^2^

Half were in the age group 21-30 years and 40% in the age group 31-40 years, with mean±SD (28.06±6.39). A similar study reports 65.2% of participants in age group 25-30 years with mean age was 27.5±4.4 years.^8^ Another similar study reports mean age 26.59± 2.87,^10^ although in both the studies age grouping is not similar majority of them are in same age group so its comparable.^8,10^ Another study also reports mean age of women 25.25±3.61 years, the finding were not consistent with our finding.^9^

The majority 60% of women were multigravida and 35% were primigravida whereas, Sitaula reported 48.8% primigravida and 51.2% multigravida in a retrospective study.^8^ The most frequent chief complaint by women was pruritus of the whole body in 70%, the finding is similar to one of the study.^2^ The weeks of gestation at diagnosis majority (90%) at 27-40 weeks with a mean of 31.38±4.56 weeks, Study reports the mean gestational age at diagnosis was 36.11±2.75 weeks,^9^ 32.53±4.68,^10^ 38 weeks 4 days^11^ respectively slightly different from our finding.^9-11^ More than 97% of case were diagnosed in third trimester which is consistent with this study.^8^ Additionally, 26.3% of women with a previous similar history of OC encountered the same risk in the present pregnancy, however, Senocak et al reports 6% of women had previous history the finding is not similar to our study the reason could be due to different settings, sample size, ethnicity.^12^

The LFT was abnormal in 83.75% had deranged. Elevated transaminases were the most frequent abnormality reported in OC. Also, the total serum bilirubin level increased by 35%, however in other similar study it was reported 29.34% bilirubin.^11^ The serum glutamic oxaloacetic transaminase (SGOT) levels were also raised, and the upper level was >250 mg/dl in 31.25% and between 100-250 mg/dl in 43.75% of patients. The serum alkaline phosphatase was 200-480 U/L in 31 (38.75%) of patients followed by 49 (61.25%) had less than 150 U/L. The findings from this study were similar to the other studies.^2,12^

Likewise, 56.25% of patients were taking tablet UDCA 150 mg twice a day followed by 32.5% were given UDCA 300 mg twice a day while 9 (11.8%) were taking UDCA 300 milligrams (mg) thrice a day.^2,10^ Similarly, 82.5% of women delivered at term 37-42 weeks of gestation with a mean of 35.51 ±3.27 weeks and the median gestational weeks was 37 similar to the finding done in another teaching hospital in Nepal.^12^

The majority of women 85% had LSCS that includes (40% emergency and 45% elective),the finding are not consistent with finding by another authors as their studies reports (46.25%),^2^ (43.4% ),^8^ (60.69%)^10^ LSCS respectively. The variation in finding could be due to different time of antenatal visit and delivery because of patient profile.^2^ In our study, only 15% of women had vaginal delivery, includes vacuum delivery 2.5%, spontaneous vaginal delivery 6.25% and 6.25% of patients had included vaginal delivery this is in alignment with finding in other studies.^2,11^ Similar studies finding reports vaginal delivery of 39.27%,^2^ 51.3%,^8^ and, 39.27%^10^ respectively. Spontaneous delivery were 10.71%.^10^

Whereas an indication of emergency LSCS in the majority 18.8% of patients was non-reassuring nonstress test (NST) 6.3% due to failed induction, 6.3% due to fetal distress and 6.3% due to meconium-stained liquor, this finding was similar to finding with various studies.^10,12^ Failed induction of labor in 33.33%, fetal distress 40%, non-progress of labor 26.66%.^10^

Similarly, the indication for elective LSCS is 20% because of previous LSCS and 10% due to cesarean delivery on maternal request (CDMR). However, 7.55% had elective LSCS due to mal-presentation including breech.^2,11^ The complications encountered following LSCS and vaginal delivery were 16.25%. Other reports showed 11.25%, 3.9%, and 3.57% and these finding are not in line with our findings.^2,8^

The birth weight of the newborn of were within the normal range (71.3%) followed by 27.5% had low birth weight. Likewise, 82.5% of them were born at term and 16.3% were born preterm. Similar studies reports preterm 13.09%,^10^ 10.67%.^13^ The majority 52.5% had an APGAR score at one minute was 8, with a mean score and SD (6.58±0.45) however other study reports less than 7 at one in 18.39%.^10^ The majority of 70.5% had a score of 9 at 5 minutes with a mean score of (7.79±0.65).^14,15^ Similar study reports 10.34% less than 7 at 5 minutes.^10^ The complications of neonates were NICU admission at 46.3%, other studies 48.75%,^2^ and 22.98%.^10^ Meconium stained liquor at 5% while 32.5%, 2 meconium aspiration syndrome at 10%,13(16.25%)^2^ in similar study. However, transient tachypnea of the newborn (TTN) at 15% and neonatal jaundice at 1.3% were other findings.

There are limitations in our study as we conducted a descriptive cross-sectional study, although it gave the prevalence of diseases, the exact cause or association were difficult to recognize and there is a chance of recall bias. So, it would be better to conduct an analytical study to identify the risk factors in settings like ours.

## CONCLUSIONS

The prevalence of OC among pregnant women is higher than other similar studies done in similar settings.
